# Empyema, acute respiratory failure, and septic shock after aspiration of a soft-shelled turtle (Pelodiscus sinensis) bone by an adult

**DOI:** 10.1186/2193-1801-3-452

**Published:** 2014-08-20

**Authors:** Gensheng Zhang, Yang Yu, Shufang Zhang, Na Li, Hao Xu, Wei Cui

**Affiliations:** Department of Critical Care Medicine, Second Affiliated Hospital, Zhejiang University School of Medicine, Hangzhou, Zhejiang 310009 P.R. China; Department of Cardiology, Binjiang Branch, Second Affiliated Hospital, Zhejiang University School of Medicine, Hangzhou, Zhejiang 310009 China; Department of Respiratory Medicine, Second Affiliated Hospital, Zhejiang University School of Medicine, Hangzhou, Zhejiang 310009 China

**Keywords:** Soft-shelled turtle bone, Foreign body aspiration, Empyema, Acute respiratory failure, Septic shock, Adult

## Abstract

**Introduction:**

The common late complications of foreign body aspiration include granulation formation, obstructive pneumonia, and atelectasis. However, a foreign body-induced pleural infection is very rare, and especially when it is not iatrogenic.

**Case description:**

A 64-year-old Chinese man was admitted to our hospital with septic shock and acute respiratory failure requiring intubation and mechanical ventilation. Computed tomography revealed multiloculated pleural effusion on the whole right side and right lung atelectasis, with enhanced thickening and calcification of pleura and a foreign body in the right intermediate bronchus. The effusion appeared as a cloudy fluid consistent with pus. A bedside bronchoscopy revealed an irregular foreign body lodged in the right intermediate bronchus. The hard bone was removed and confirmed to be a soft-shelled turtle bone. A final diagnosis of foreign body-induced empyema, acute respiratory failure, and septic shock was confirmed. The patient showed good recovery after completing a course of broad-spectrum antibiotics and undergoing chest tube drainage.

**Discussion and evaluation:**

Although empyema has been reported previously as a rare complication of long-term retention of an aspirated foreign body, no case has been observed that was as serious as our current patient. In addition, a foreign body aspiration by a soft-shelled turtle bone was never reported before.

**Conclusions:**

For the first time, we describe the successful treatment of an adult patient presenting with empyema, accompanied by serious conditions of acute respiratory failure and septic shock induced by aspiration of a soft-shelled turtle bone. Clinicians should consider the possibility of non-iatrogenic foreign body-induced empyema with acute onset of respiratory failure, when a patient’s symptoms cannot be attributed to an alternative obvious cause.

## Background

Although the clinical manifestations and management of foreign body (FB) aspiration in children and adults have been well summarized in several previous case series (Baharloo et al. 1999; Dong et al. 2012; Nguyen et al. 2011), pleural infection following inhalation of a non-iatrogenic FB is a very rare complication, and only a few case relevant case reports have been published (Ota and Kawai 2012; Dilege et al. 2002; Behl et al. 2008; Asadi Gharabaghi et al. 2012; Schembri et al. 2003). Acute onset of respiratory distress and severe sepsis caused by empyema after inhalation of a foreign body has never been reported. Herein, we describe for the first time a case of FB-induced pleural empyema presenting with accompanying acute respiratory failure and septic shock following chronic retention of an aspirated soft-shelled turtle (Pelodiscus sinensis) bone. Following diagnosis and treatment, the patient demonstrated a good recovery.

## Case description

**Figure 1:**
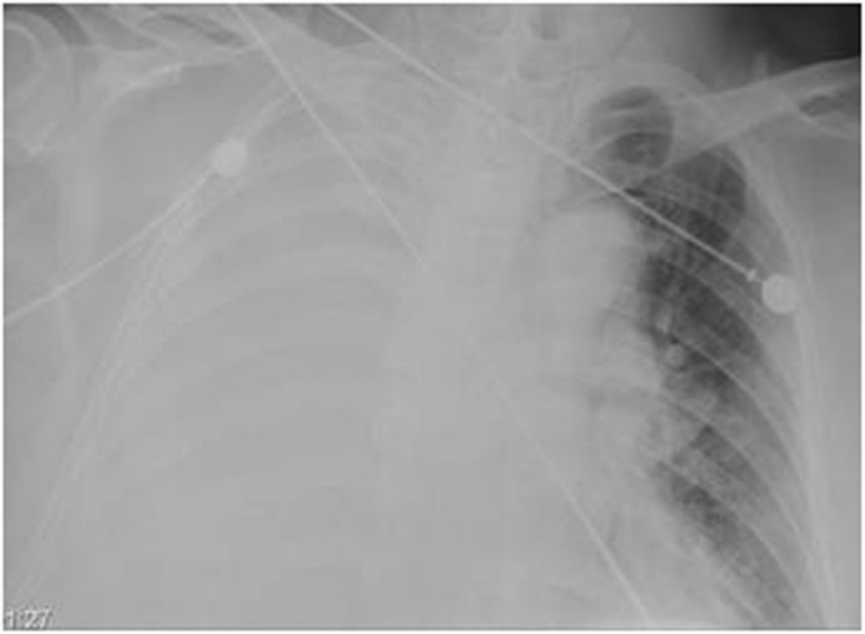
**Chest radiograph showing a whole consolidation and**/**or pleural effusion of the right lung.**

**Figure 2:**
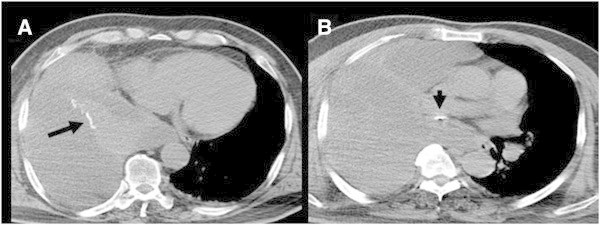
**Computed tomography scan showing a large encapsulated collection with consolidation of the whole right lung,** **enhanced thickening and calcification of pleura** **(black arrow in A),** **and a foreign body in the right intermediate bronchus** **(black arrow in B.)**

A 64-year-old Chinese man was admitted to the emergency department of our hospital with chest pain, a productive cough, and three days of untreated progressive dyspnea. Five years earlier, he had been admitted to a local hospital for treatment of cerebral hemorrhage, right rib fractures, large pleural effusion, and a left femur fracture resulting from a traffic accident. Although he showed good recovery, the patient continued to have a chronic cough and intermittent chest tightness. Upon initial evaluation in the emergency room of our hospital, the patient was drowsy, and his body temperature, pulse rate, blood pressure, and respiratory rate, were 36.8°C, 110/min, 86/50 mmHg, and 32/min, respectively. An analysis of arterial blood gases in room air showed pH 7.52, PCO_2_ 31 mmHg, PO_2_ 52 mmHg, SaO_2_ 85%, and a high level of lactate (4.6 mmol/L). A chest radiograph showed right lung consolidation and possible pleural effusion (Figure 1). Computed tomography (CT) revealed multiloculated pleural effusion on the whole right side and right lung atelectasis, with an enhanced thickening and calcification of pleura, and a foreign body in the right intermediate bronchus (Figure 2). The patient’s condition was rapidly deteriorating, and he was intubated and supported by mechanical ventilation. Following initial hemodynamic stabilization, the patient was transferred to the intensive care unit (ICU).

On physical examination, the patient was afebrile with a blood pressure of 108/67 mmHg under the support of intravenous norepinephrine (6 μg/kg/min). On auscultation, moist rales and wheezing sounds were heard in both lung fields, with decreased breath sounds in the right lower lung. A complete blood count showed an elevated white count of 27.7 × 10^9^/L with a high percentage (94.6%) of neutrophils. Concentrations of C-reactive protein (patient level 433.1 mg/L v.s. normal range of 0 ~ 8 mg/L) and serum procalcitonin (patient level 5.44 ng/mL v.s. normal range of 0 ~ 0.05 ng/mL) were markedly elevated. The patient was supported with mechanical ventilation. Chest tube drainage was performed under ultrasound guidance, and the effusion appeared as a cloudy fluid consistent with pus, with total nucleated cells of 920 × 10^6^/L. Additionally, the effusion was positive in Rivalta’s test. The results of a Gram stain and acid fast stain for the fluid were negative.

**Figure 3:**
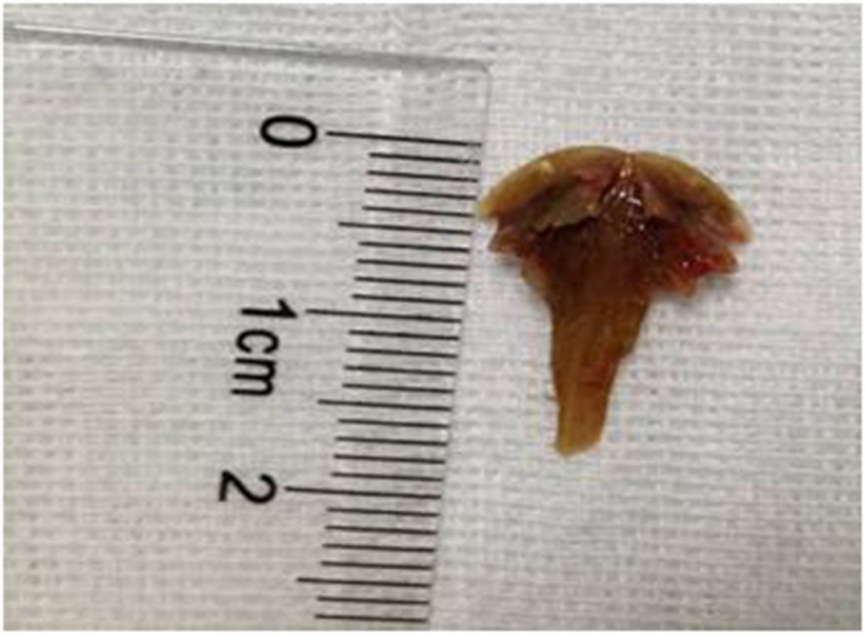
**Extracted T**-**shaped soft**-**shelled turtle bone measuring** ~ **1.3** × **1.7 cm.**

Based on these findings, a diagnosis of empyema accompanied by acute respiratory failure and septic shock was considered. Samples of pleural effusion, blood and sputum were sent for aerobic and anaerobic cultures, and empirical treatment with broad-spectrum antibiotics (imipenem-cilastatin (0.5 g, q6h) and linezolid (0.6 g, q12h)) was initiated. Resuscitation with fluids was initiated based on guidelines in the Surviving Sepsis Campaign, 2012 (Dellinger et al. 2013). On the second day of treatment, the patient’s hemodynamic parameters had stabilized and the intravenous norepinephrine was withdrawn. A flexible bronchoscopy performed at bedside revealed an irregular foreign body lodged in the right intermediate bronchus. As the size was too large to pass through the tracheal tube (internal diameter = 8.0 mm) and the texture was too hard to clamp or cut into small pieces, repeated attempts to remove the foreign body with forceps were unsuccessful. We then attempted and were successful in removing the foreign body using a 1.9 mm cryotherapy probe provided with the ERBE cryotherapy system (Erbokryo CA, ERBE Cryosurgery, Tubingen, Germany). The tracheal tube was removed in conjuction with the foreign body. The foreign body was a hard T-shaped bone measuring ~ 1.3 × 1.7 cm (Figure 3). After removing the bone, the patient was re-intubated for mechanical ventilation under the guidance of bronchoscopy.

**Figure 4:**
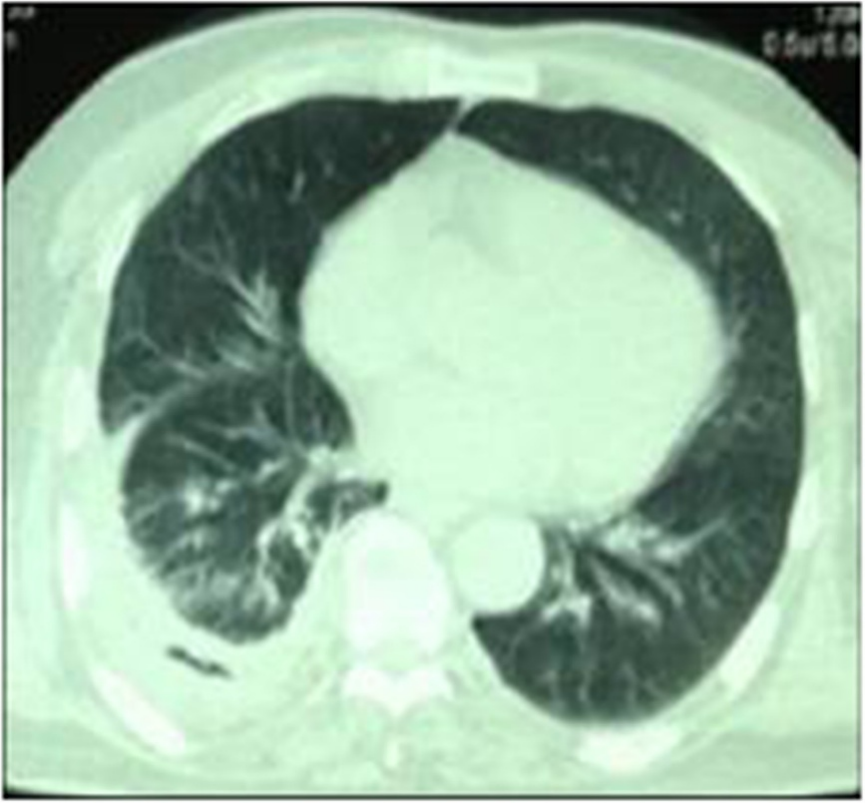
**Follow**-**up computed tomography conducted one month after extraction of the foreign body showing almost complete absorption of the right pleural empyema**.

Following FB removal, the initial antibiotics treatment was continued for five days, even though the culture results of blood, sputum, and pleural effusion were negative. The patient rapidly improved and was successfully extubated one week later. When asked about the circumstances of the FB aspiration, the patient stated that 3 months earlier, he had choked while eating the meat of a soft-shelled turtle; however, he did not see a doctor or take medicine at any time. Thus, the foreign body was confirmed as a bone from a soft-shelled turtle. The patient recovered uneventfully and was discharged from the hospital 4 weeks after admission. A follow-up chest CT scan conducted one month later showed almost complete absorption of the pleural effusion (Figure 4). The patient was then followed up for 6 additional months, and showed an uneventful recovery.

## Discussion

While FB aspiration is relatively rare in adults, some adults have significant risk factors including various neurological and neuromuscular diseases, or a history of head trauma; such as in the case of our current patient who had a previous history of cerebral hemorrhage caused by a traffic accident. In contrast to children, “penetration syndrome” (a sudden onset of choking and intractable cough) in adults is extremely unusual, and most cases present with non-specific symptoms such as a chronic cough or wheezing (Baharloo et al. 1999; Dong et al. 2012; Nguyen et al. 2011). Prior to being correctly diagnosed, these patients are often incorrectly diagnosed with other pulmonary diseases such as upper respiratory tract infections, simple pneumonia, etc. (Dong et al. 2012; Nguyen et al. 2011). Some studies (Baharloo et al. 1999; Dong et al. 2012; Nguyen et al. 2011) have reported delayed diagnoses of FB aspiration for time periods ranging from 3 h to 15 months. Such patients are usually hospitalized as a result of mild/serious complications of long-term retention of a FB; e.g., recurrent pneumonia. However, no case has been reported that was as serious as our current patient who presented with acute respiratory failure and septic shock caused by whole right-side empyema following non-iatrogenic FB aspiration.

The incidence of complications resulting from long-term retention of an aspirated foreign body is very high, and ranges from 62.9% to 79.5% (Dong et al. 2012; Debeljak et al. 1999). In the largest study of FB aspiration ever conducted in adults, the most frequently reported complications were granulation formation (76.5%), obstructive pneumonia (22.0%), bleeding (14.5%), atelectasis (10.0%) and endobronchial stenotic scarring (8.0%) (Dong et al. 2012). Only a few cases of non-iatrogenic FB-induced empyema have been reported (Ota and Kawai 2012; Dilege et al. 2002; Behl et al. 2008; Asadi Gharabaghi et al. 2012; Schembri et al. 2003). Similar to other long-term complications, the major symptoms displayed by adults with FB-induced empyema include a productive cough, fever, chest pain, shortness of breath, and dyspnea (Ota and Kawai 2012; Dilege et al. 2002; Behl et al. 2008; Asadi Gharabaghi et al. 2012; Schembri et al. 2003). Additionally, an extended period of time may be required for empyema to develop as a result of FB aspiration. In our case, empyema developed after the patient had inhaled a foreign body only three months earlier, which was faster than the 8 months to 4 years (Dilege et al. 2002; Asadi Gharabaghi et al. 2012) or even > 30 years (Schembri et al. 2003) reported in previous studies. In addition, our patient developed a whole right-side empyema, rather than a local empyema described in previous cases (Dilege et al. 2002; Behl et al. 2008; Asadi Gharabaghi et al. 2012; Schembri et al. 2003). Finally, the enhanced thickening of pleura caused by previous right rib fractures 5 years earlier may have contributed to the rapid formation of whole right empyema in our patient, by hampering the normal absorption of pleural effusion.

Most empyemas result from bacterial suppuration in adjacent structures (such as the lung, mediastinum or chest wall), following an invasive procedure (i.e., thoracentesis) or chest injury. However, the mechanisms for non-iatrogenic FB-induced empyema are not well understood (Dilege et al. 2002). Post-obstructive pneumonia is the main mechanism by which foreign body aspiration causes empyema. Late complications of foreign body aspiration (e.g., bronchopleural fistulas and a secondary empyema) usually stem from post-obstructive pneumonia or an obstructive infection. The direct obstruction produced by aspiration of a foreign body, and any subsequent inflammatory response or secondary infection, may result in a post-obstructive pneumonia. Additionally, the unidirectional movement of an aspirated FB such as a T-shaped bone may facilitate its migration from the bronchus into the pleural space (Dilege et al. 2002; Schembri et al. 2003). This occurrence might facilitate the development of a pulmonary pleural fistula and/or empyema under conditions of an obstructive infection. Aside from the common aerobic organisms such as Streptococcus spp. and staphylococci which are mentioned as the main pathogens causing pleural infections in the British Thoracic Society Pleural Disease Guideline 2010 (Davies et al. 2010), the culture results of pleural fluid resulting from a FB-induced pleural empyema are an anaerobic agent (e.g., Campylobacter) or negative (Dilege et al. 2002; Behl et al. 2008). This may be explained by the possible anaerobic environment created by direct obstruction with a FB and/or the formation of a granuloma induced by chronic inflammation.

One limitation to the current study is that we did not identify a reason for the rapid and serious empyema which suddenly developed three months after FB aspiration. In addition to the patient’s reluctance to see a doctor, the following possible explanations should be considered: (a) The patient may have tolerated mild to moderate dyspnea during the development of empyema as a long-term adaptation to chronic cough and intermittent chest tightness which developed after the traffic accident five years earlier. (b) Although the culture results for the pleural effusion were negative, infection could not the totally excluded due to the possibility of anaerobic infection. In our case, the patient was afebrile before and even after the occurrence of septic shock, and this is consistent with the features of anaerobic infections which develop with insidious clinical onset and less fever (Bartlett et al. 1974). The lack of early and obvious symptoms may have caused the patient to delay seeking medical attention.

## Conclusion

We described the successful treatment of an adult patient who presented at our hospital with serious conditions of empyema, acute respiratory failure, and septic shock induced by aspiration of a soft-shelled turtle bone. Clinicians should consider the possibility of non-iatrogenic FB-induced empyema accompanied by acute onset of respiratory failure when diagnosing and treating patients who present with such conditions, without an obvious cause (e.g., infection of adjacent structures or a direct chest injury).

## Abbreviations

FB: Foreign body
CT: Computed tomography
ICU: Intensive care unit.
